# Analyzing the impact of 900 MHz EMF short-term exposure to the expression of 667 miRNAs in human peripheral blood cells

**DOI:** 10.1038/s41598-021-82278-1

**Published:** 2021-02-24

**Authors:** Andreas Lamkowski, Matthias Kreitlow, Jörg Radunz, Martin Willenbockel, Marcus Stiemer, Lars Ole Fichte, Carl Friedrich Rädel, Matthäus Majewski, Patrick Ostheim, Matthias Port, Michael Abend

**Affiliations:** 1 Bundeswehr Institute of Radiobiology Affiliated To University Ulm, Neuherbergstr. 11, 80937 Munich, Germany; 2 Bundeswehr Research Institute for Protective Technologies and CBRN Protection, Humboldtstraße 100, 29633 Munster, Germany; 3 Bundeswehr, Helmut Schmidt University/University of the Federal Armed Forces, Holstenhofweg 85, 22043 Hamburg, Germany

**Keywords:** Molecular biology, miRNAs

## Abstract

More than ever before, people around the world are frequently exposed to different sections of the electromagnetic spectrum, mainly emitted from wireless modern communication technologies. Especially, the level of knowledge on non-thermal biological EMF effects remains controversial. New technologies allow for a more detailed detection of non-coding RNAs which affect the post-transcriptional control. Such method shall be applied in this work to investigate the response of human blood cells to electromagnetic irradiation. In this ex vivo in vitro study, we exposed peripheral blood cells from 5 male donors to a continuous wave of 900 MHz EMF for 0, 30, 60 and 90 min. Significant micro RNA (miRNA) expression changes (*p* ≤ 0.05) above or below the SHAM exposed samples were evaluated using a quantitative real time PCR platform for simultaneous detection of 667 miRNAs called low density array. Only significant miRNA expression changes which were detectable in at least 60% of the samples per exposure group were analyzed. The results were compared with data from room temperature + 2 °C (**RT** + 2 °C) samples (here referred to as hyperthermia) to exclude miRNA expression altered by hyperthermia. The validation study by using the same donors and study design was performed after an interval of 2 years. When analyzing a total of 667 miRNAs during the screening study, 2 promising candidate miRNAs were identified, which were down regulated almost twice and showed a complete separation from the unexposed control group (miR-194 at 30 min and miR-939 at 60 min). The *p*-values even survived the Bonferroni correction for multiple comparisons (*p* = 0.0007 and *p* = 0.004, respectively). None of these miRNAs were expressed at a second time point after EMF exposure. Following an alternative analysis approach, we examined for miRNAs revealing an expected significant association of differential miRNA expression with the dose-time EMF exposure product, separately for each donor. Donors 2 and 3 revealed 11 and 10 miRNA species being significantly associated with EMF exposure which differed significantly from the other donors showing a minor number of differentially expressed miRNAs and could identify donors 2 and 3 as particularly EMF-responsive. The measurements were repeated after 2 years. The number of expressed/non-expressed miRNAs was almost similar (97.4%), but neither the number nor the previously differentially expressed miRNAs could be reproduced. Our data neither support evidence of early changes at miRNA expression level in human whole blood cells after 900 MHz EMF exposure nor the identification of EMF-responsive individuals.

## Introduction

The targeted utilization of radiofrequency electromagnetic fields (EMF) is the basis for all wireless communication technologies and cannot be substituted within an era of steady information and data exchange. Due to the upcoming demand for higher channel capacities during mobile data transmission, many countries have started to upgrade networks with the 5G standard. Nevertheless, former mobile network standards are continued to be operated that lead to increasing total EMF emissions with a particular focus in urban areas. Extensive studies searching for athermal EMF effects have been carried out in recent decades and health impairment initiated by EMF at the molecular level was not reproducibly confirmed. However, the principal idea of specific biological responses following EMF cannot be excluded, because many biological structures within a cell are susceptible for electromagnetic interactions due to their electric charged state and even by using their electric potential during vital processes. If cells sense alterations after EMF exposure this might lead to a response by activation of the transcriptome. Here, protein coding genes will be activated by increasing the gene copy number and corresponding proteins will be synthesized leading to certain biological responses such as cell cycle control, apoptosis or DNA repair. These protein coding genes are controlled by so called micro ribonucleic acids (miRNAs). These miRNAs are highly conserved short sequences acting as essential control elements of gene regulation which has been shown in numerous studies. Based on the current state of knowledge, up to 60% of protein coding genes of the human genome are regulated by complex interactions involving miRNAs^1^. MiRNA contribution is common in various cellular stress responses such as regulation of coding genes with involvement, inter alia, in apoptosis, cell cycle and DNA repair^2^. In this connection, Ivanovska et al. 2008 demonstrated that miR-106b promotes cell-cycle progression^3^ whereas miR-24 was identified to control the H2AX expression^4^ which is the key protein in the DNA damage response^5^.

It has also been shown that some miRNAs affect p53-dependent regulation^6,7^. Accumulating indications have been found that miRNAs are regulators of immune response and inflammation^8^. Other authors proved that miRNAs act as crucial elements in posttranscriptional control of erythroid cells^9–11^.

These and many other studies highlight the pivotal role of miRNAs and raised questions regarding an involvement in an EMF specific response in order to stabilize cellular homeostasis.

A few previous studies have already examined for a potential association between extremely low frequency electromagnetic fields and miRNA expression. It has been speculated that the 50 Hz electric power supply with magnetic field components exceeding 2 mT is sufficient to induce miR-26b-5p expression changes in GC-2-cells^12^. Our study was focused on the screening of miRNA expression patterns as a specific biological response in peripheral human blood cells after EMF exposure and follows an agnostic approach since reproducible athermal EMF effects could not be demonstrated in previous studies. We established a whole blood ex vivo in vitro model which were exposed to 900 MHz EMF for 30, 60 und 90 min^13^ and measured miRNA expressions by qRT-PCR.

## Material and methods

### Two-stage study design

**Figure 1:**
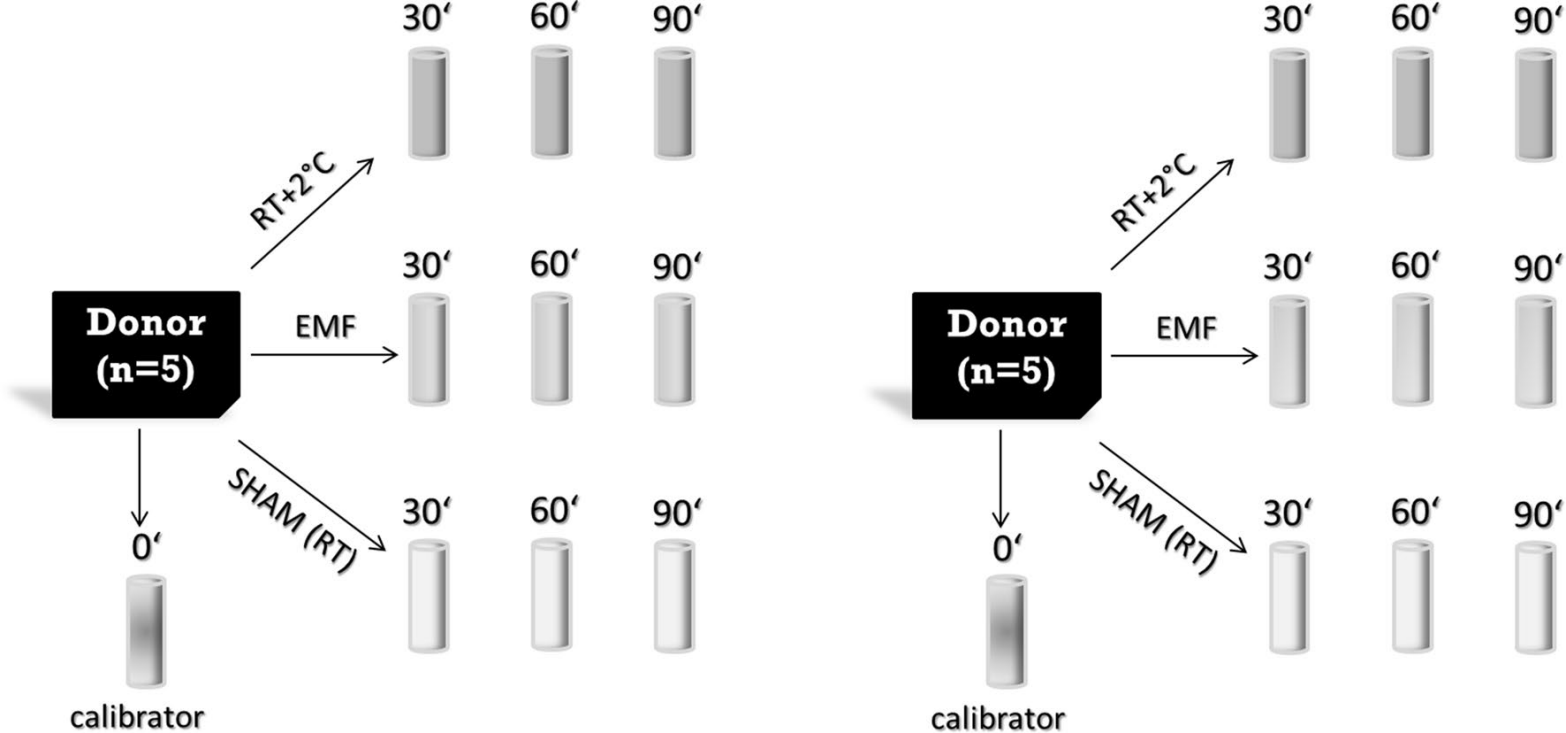
10 EDTA tubes were drawn from each donor (n = 5) and divided in the 3 different exposure situations (SHAM, 900 MHz EMF and RT + 2 °C). Additionally, 1 sample from each donor with 0 min exposure was used as the calibrator sample. Since the EMF exposure occurs over time (30, 60 and 90 min) and gene expression changes already arise through cultivation, the controls (RT + 2° C and SHAM) must also be cultivated for 30, 60 and 90 min. The experiment was performed in 2016 and repeated including the same donors in a reproducible manner in 2018.

In 2016 we performed a miRNA screening study using EMF exposed whole blood samples from 5 donors. This study was carried out exactly the same way in 2018 and, thus, represents an independent validation of our findings 2 years ago (Fig. 1).

### Donors and ex-vivo model

In this study, we used again our ex-vivo in vitro model with whole blood collected in EDTA (Ethylene Diamine Tetraacetic Acid) tubes as originally described in Lamkowski et al. 2018^13^. Before the start of the study, the donors were asked about the existence of acute or chronic illnesses, alcohol consumption, operations, accidents, vaccinations and nicotine abuse. In addition, peripheral blood counts were performed in order to rule out leukocytoses or leukopenias as well as relevant shifts in the leukocyte subpopulations in the differential blood count. There were no relevant findings among the study participants that would have made their exclusion from the study necessary. Each of the five healthy male donors aged 30–40 years at the time of biosampling provided one EDTA tube for a differential blood cell count and 10 EDTA tubes were dedicated for whole blood gene expression analysis. These EDTA tubes were either SHAM or EMF exposed or treated with a temperature of 2 °C above room temperature (referred to as hyperthermia). An up to threefold increase in EMF exposure (exposure-time product) was realized by extending exposure time from 30 min (1xEMF) over 60 min (2xEMF) and 90 min (3xEMF) without changing the EMF-field parameter. This was necessary in order to preserve a homogenous EMF exposure of the EDTA tubes. As a result one EDTA tube served as unexposed control and 3 × 3 EDTA tubes became exposed over 30 min, 60 min and 90 min either with EMF (3 tubes), SHAM (3 tubes) or 2 °C above room temperature (hyperthermia, 3 tubes) as demonstrated in Lamkowski et al. 2018^13^ (see also Fig. 1).

After phlebotomy all 10 EDTA tubes were cooled down in a water bath adjusted to room temperature (RT) to quickly accomplish equal baseline conditions (RT = 21.6 ± 0.5 °C in 2018). Afterwards, one of the EDTA tubes representing the unexposed control was directly pipetted to a PAXgene tube (BD Diagnostics, PreAnalytiX GmbH, Hombrechtikon, Switzerland) to inhibit Ribonucleases. PaxGene tubes contain chemical additives that stabilize the RNA molecules and prepare a later purification. The PaxGene system is therefore used to preserve the RNA expression in cells that were previously cultivated under EMF or SHAM exposure conditions. Three EDTA tubes were SHAM exposed at room temperature conditions. Three other EDTA tubes were simultaneously positioned and exposed in the EMF chamber. The next three samples were heated to 2 °C above room temperature level, while keeping them in a water bath. After 30, 60 and 90 min, we draw one tube from each exposure group (EMF, SHAM, 2 °C above room temperature) and transferred each EDTA whole blood into a separate PAXgene tube to maintain RNA integrity. PAXgene tubes were hold at room temperature overnight and stored at − 20 °C until used.

### Exposition and dosimetry

The experimental setup consists of an open wave guide which is apart from the missing side walls built as a Mini Transverse Electromagnetic Cell (Mini-TEM cell), a water bath and a flat surface for room temperature samples. Since an equivalent electric field distribution as in a Mini-TEM cell is obtained, we will refer to the device as a Mini-TEM cell in the sequel^13^. All these areas were continuously monitored with respect to temperature by focusing an infra-red camera towards the samples. The Mini-TEM cell was operated with 10 W input power establishing an electric field strength of around 134 to 145 V/m for the main component. The blood tubes were oriented in parallel to the electric field to maximize the coupling. The applied signal to the cell was a continuous wave (CW) with a frequency of 900 MHz. The adjustable temperature regulated water bath ensured a rapid and homogeneous heat control of all samples during 1 Hz shaking motion. The blood tubes were cooled down to room temperature immediately after blood collection during a mean period of 6 min and subsequently referred to the experimental groups. Three samples from each donor *(n* = *3 (time points)* × *5 (donors)* = *15 EMF samples)* were placed in the exposition chamber in spatial orientation parallel to the electric field lines. Three sham samples from each donor *(n* = *3 (time points)* × *5 (donors)* = *15 sham samples)* were positioned outside of the Mini-TEM cell while field measurement showed an exposition lower than 5 V/m there. Furthermore, three samples *(n* = *3 (time points)* × *5 (donors)* = *15 RT* + *2 °C samples)* were heated to 2 °C above RT under consistent measurements with the infra-red camera (frame rate of 0.05 pictures/s) and a thermocouple. In order to analyze the camera data, regions of interest (ROI) were defined and min, max and mean values acquired. Five ROIs of similar size were specified focusing on the background of the Mini-TEM cell (polystyrene of sample holder), the water bath and the exposed blood tubes. The assumption that the field dose applied to the samples has not significantly changed in the second study relies on a careful reproduction of the experimental setup. During the validation study, the exact experimental set-up was reproduced on the basis of detailed photo documentation and logging, which were collected during the screening study. The same experimental components (e.g. EMF source, amplifier, waveguide, etc.) were used in both the screening study (2016) and the validation study (2018). The operational personnel were also the same for both studies. All possible other causes that may lead to a change in the electromagnetic field inside the cell have been excluded, e.g. the amplifier settings have been identically reproduced. Additional electromagnetic reflections, that could be a consequence of changed impedances due to geometrical deviations of the waveguides or the Micro-TEM cell, particularly its termination impedance, have been excluded by a thorough repetition of all geometrical adjustments. This includes the samples in the cell, whose composition and size has carefully been reproduced. Particularly the power balance, and hence, the specific absorbed power (SAR, in W/kg), which is the relevant notion of a dose in this frequency regime, remain unchanged.

In addition, in the report on the first study^13^, it has been carefully argued that only moderate variations of the irradiation dose in terms of the specific absorption rate (SAR) arise due to different sample positions (e.g. to shadowing or local deviation of field lines). All values determined for the first period of irradiation stay between 7 and 10 W/kg. Further, only a moderate increase of the dose has been observed for the remaining samples after the removal of samples after 30 min and 60 min. The highest value applied on a sample staying alone after 60 min was 13.27 W/kg.

Apart from validation of spatial inhomogeneity and temporal dose changes, an estimate of the SAR is essential to categorize the exposure situation and for comparison to other studies. To this end, a method to estimate the applied SAR has been proposed in^13^ and applied to the results of the 2016 study: Basically, the SAR can be determined from temperature measurements. In case of a homogeneously irradiated small sample (such that heat conduction can be neglected) with specific heat capacity $c$, surface size $A$, heat transfer coefficient $\alpha$, mass $m$ and time-variant ambient temperature ${T}_{\mathrm{ENV}}\left(t\right)$, the SAR ${f}_{\mathrm{SAR}}$ fulfils the linear first order inhomogeneous ordinary differential equation

1 $$\frac{dT}{dt}\left(t\right)+\frac{\alpha A}{cm}T\left(t\right)= \frac{{f}_{\mathrm{SAR}}}{c}+\frac{\alpha A}{cm}{T}_{\mathrm{ENV}}\left(t\right)$$

which stems from a power balance of the irradiated sample accounting for power supply due to the electromagnetic field and to power transfer over its surface proportional to $- \alpha A\left({T\left(t\right)-T}_{\mathrm{ENV}}\left(t\right)\right)$. The latter contribution causes exponential cooling for ${T\left(t\right)>T}_{\mathrm{ENV}}\left(t\right)$ and, hence works against the field exposure, while it supports exponential heating if ${T\left(t\right)<T}_{\mathrm{ENV}}\left(t\right)$. With $w=\frac{\alpha A}{cm}$, $\gamma =\frac{{f}_{\mathrm{SAR}}}{cw}$, and ${T}_{0}=T(0)$ the formal solution of (1) reads

2 $$T\left(t\right)=\gamma +{e}^{-wt}\underset{0}{\overset{t}{\int }}{T}_{\mathrm{ENV}}\left(t{^{\prime}}\right){e}^{w{t}^{^{\prime}}} \mathrm{d}t{^{\prime}}+ \left({T}_{0}-\gamma \right){e}^{-wt}$$

There are two measurement approaches to exploit (2) for SAR determination: temperature control and temperature measurement. Temperature control requires to keep the environmental temperature ${T}_{\mathrm{ENV}}$ constant reducing (2) to

3 $$T\left(t\right)=\gamma +{T}_{\mathrm{ENV}}+ \left(\left({T}_{0}-{T}_{\mathrm{ENV}}\right)-\gamma \right){e}^{-wt}$$

In addition, the initial temperature of the samples is set to the ambient temperature, i.e., ${T}_{0}={T}_{\mathrm{ENV}}$. Under these conditions, ${f}_{\mathrm{SAR}}=cw\gamma =cT{^{\prime}}(0)$. The SAR reconstruction in^13^ has been carried out for such a setting. This approach is justified by the clear separation between background temperature (about 19.7 °C) and sample temperature and the relatively good adjustment of the samples’ initial temperature. In the second study, the measured temperature background was higher (> 21.6 °C) and interfered with the samples’ temperatures curves. To avoid the numerically delicate determination of $T{^{\prime}}(0)$ from the measured temperature data, $w$ and $\gamma$, and, hence, ${f}_{\mathrm{SAR}}$ have been determined by a fit of the model curve (3). Details on the mathematical optimization method (provided by the mathematical software MATLAB) are given in^13^. In contrast, temperature measurement allows for changes of the environmental temperature ${T}_{\mathrm{ENV}}\left(t\right)$, and ${T}_{0}=T(0)$ is not adjusted. Both quantities are measured. In case of an exponential decay of the background temperature

$${T}_{\mathrm{ENV}}\left(t\right)={T}_{\mathrm{ENV}}\left(\infty \right)+ \left({T}_{\mathrm{ENV}}\left(0\right)-{T}_{\mathrm{ENV}}\left(\infty \right)\right){e}^{-\alpha t}$$

in the Mini-TEM cell, which has been observed and considered as significant in the temperature results of the second study, the integral in (2) can formally be computed. After $\alpha$ and ${T}_{\mathrm{ENV}}\left(\infty \right)$ have been identified by a non-linear least-square fit of the temperature model to the data measured for ${T}_{\mathrm{ENV}}$, these are inserted in (2), and $w$, $\gamma$, and ${T}_{0}$ are assessed by a second fit such that ${f}_{\mathrm{SAR}}= \gamma cw$ can be computed. To compare the field dose applied to the samples in the first and in the second study, the assessment of the SAR values for the first study has been repeated with the method that accounts for varying background temperature. The results are presented in Table 1 (left hand side in each position). As a general trend most SAR values determined with the new method are 10–20% lower. However, the principal dimension and particularly their relative magnitudes remain the same. For this study, only the first phase with all samples present has been considered for SAR reconstruction, since further changes during the whole exposition time have already been studied in^13^.

**Table 1:** Assessment of the SAR (in W/kg) from the measured temperature data for both studies in 2016 and 2018, all donors (P1–P5), and all sample positions in the Mini-TEM cell during the first 30 min of field exposition.

	Top (30 min) exp-model/linear		Mid (90 min) exp-model/linear		Bottom (60 min) exp-model/linear	
P1 (in 2016)	7.3 ± 0.5	5.4 ± 0.6	8.5 ± 0.6	6.4 ± 0.6	6.9 ± 0.7	4.6 ± 6.0
P1 (in 2018)	4.8 ± 0.1	8.2 ± 1.8	5.4 ± 0.1	12.1 ± 0.7	4.8 ± 0.1	9.7 ± 0.8
P2 (in 2016)	9.2 ± 0.3	10.0 ± 1.6	7.2 ± 0.3	6.0 ± 0.3	6.9 ± 0.2	10.1 ± 0.8
P2 (in 2018)	6.2 ± 0.2	5.9 ± 0.5	6.7 ± 0.2	10.6 ± 1.2	6.0 ± 0.2	10.0 ± 0.4
P3 (in 2016)	fit failed	0.0 ± 3.2	7.1 ± 0.6	4.0 ± 0.5	fit failed	0.8 ± 0.8
P3 (in 2018)	7.1 ± 0.2	7.8 ± 1.4	7.2 ± 0.2	13.0 ± 0.6	6.0 ± 0.2	5.3 ± 1.6
P4 (in 2016)	8.5 ± 0.2	18.1 ± 2.1	6.8 ± 0.2	14.8 ± 1.6	7.4 ± 0.3	11.0 ± 1.5
P4 (in 2018)	2.3 ± 0.1	15.2 ± 0.4	0.7 ± 0.05	16.5 ± 0.5	0	16.5 ± 0.8
P5 (in 2016)	8.6 ± 0.6	8.5 ± 0.2	8.9 ± 0.7	7.1 ± 0.3	8.3 ± 0.9	5.4 ± 0.4
P5 (in 2018)	6.3 ± 0.1	12.2 ± 1.4	5.6 ± 0.1	17.0 ± 0.7	5.8 ± 0.1	14.6 ± 0.5

In each position, the first number refers to the exponential model with preceding fit of the temperature background and the second number to a linear fit about $t=0$.

${\mathrm{T}}_{\mathrm{ENV}}\left(\mathrm{t}\right)$ Both ways of SAR reconstruction suffer from a serious drawback: From an experimental point of view temperature measurement and temperature control are quite difficult. While an infrared camera provides a stable measurement of temperature differences with an accuracy of typically less than ± 0.1 °C, measurement of absolute temperatures requires frequent and careful calibration to compensate for the different surface emissivity and the influence of reflected infrared radiation that superposes the thermal radiation of a body. Further accuracy issues may result from an inhomogeneous infrared radiation field over the chosen regions of interest. The temperature curves measured in the two studies indicate that two problems have occurred: Firstly, the origin of the temperature scale for the background has an offset compared with that of the sample. Secondly, the measurement data show that the initial temperature of the samples could not be adjusted sufficiently accurate: While for the first study the samples were slightly too hot at $t=0$, in the second study, the initial temperature of the samples was adjusted too cold. This becomes obvious in numerically computing

$$c{T}^{^{\prime}}\left(0\right)={f}_{\mathrm{SAR}}+cw\left({T}_{\mathrm{ENV}}\left(0\right)-T\left(0\right)\right)$$

and comparing it with an identification method that assumes ${T}_{\mathrm{ENV}}\left(0\right)-T\left(0\right)=0$. For the results in the first study the additional term $cw\left({T}_{\mathrm{ENV}}\left(0\right)-T\left(0\right)\right)$ leads in most cases to an underestimation of ${f}_{\mathrm{SAR}}$, in the second study ${f}_{\mathrm{SAR}}$ was strongly overestimated by $c{T}^{^{\prime}}\left(0\right)$. In Table 1 these figures are added to the result of a fit to (1) using an exponential model for ${T}_{\mathrm{ENV}}$.

Applying the new method of SAR estimation to the results of the second study yields due to the overestimation of the ambient temperature a generally lower SAR with a typical reduction of about 1/3 (Table 1). The real uncertainties are higher than the given values which only account for the standard deviation of the temperature data and of the specific heat capacity $\mathrm{c}= \left(3594\pm 16\right) \frac{\mathrm{J}}{\mathrm{kg K}}$ of blood^14^. They do not comprise uncertainties in the parameters determined for the background temperature or the spatial variation of the temperature measured in the observed regions of interest. Moreover, errors due to a systematically wrong measured ambient temperature are not comprised. The assumption that the samples have been adjusted to the ambient temperature in the beginning of the experiments (in case of temperature control the absolute temperature need not be known for a good fit) is also not justified for the second study. The resulting SAR determined from the linearized model (3) are hence overestimated and are given in Table 1 on the right hand side in each position.

In spite of all these methodical problems related to sufficient accurate measurements of absolute temperatures on different surfaces, the recorded data support that the real SAR probably took values between the upper bound for whole body exposure for occupationally exposure (4 W/kg) in this frequency regime and the upper bound for localized exposure of limbs for this group (20 W/kg) and is, hence, chosen from a regime where so far no relevant biological effects have been reported. This is exactly the region of electromagnetic dose that is the most interesting for searching for non-thermal effects of electromagnetic fields.

### RNA extraction and quality control

The following methodology of RNA extraction and quality control has already been described by Abend et al. 2014^15^. After thawing, washing and centrifugation, cells (including RNAs from the serum) in the pellet of the PAXgene tubes became lysed (Proteinase K) followed by adding Lysis/Binding Solution taken from the mirVana Kit (Life Technologies, Darmstadt, Germany). Using the mirVana kit the total RNA including small RNA species was isolated by employing a Phenol–Chloroform RNA precipitation followed by a silica membrane method. After several washing procedures DNA residuals became digested on the membrane (RNAse free DNAse Set, Qiagen, Hilden, Germany), and subsequently washed, before RNA was eluted in a collection tube and frozen at − 20 °C. Quality and quantity of isolated total RNA were measured spectrophotometrically (NanoDrop, PeqLab Biotechnology, Erlangen, Germany). RNA integrity was assessed by the 2100 Agilent Bioanalyzer (Life Science Group, Penzberg, Germany) and DNA contamination was controlled by conventional polymerase chain reaction (**PCR**) using beta-actin primer. We used only RNA specimens with a ratio of A_260_/A_280_ ≥ 2.0 (Nanodrop) and RNA integrity number (RIN) ≥ 7.5 for further analyses.

### Quantitative real time PCR (qRT-PCR)

The following methodology of qRT-PCR has already been described by Abend et al. 2014^15^. For qRT-PCR we employed so called low density arrays (LDA) covering altogether 667 different miRNA species which can be detected simultaneously using two cards with each of them allowing 384 separate qRT-PCRs (LDA A and B). Aliquots from each RNA sample (2 µg/LDA) were reversely transcribed without preamplification over three hours using the so called “*Megaplex pools without preamplification protocol for miRNAs expression analysis protocol*”. Since two different LDAs existed it was necessary to create two kinds of cDNAs suitable for each of both LDAs using different sets of primers. In a second step, the whole template cDNA and 450 µl 2 × RT-PCR master mix were adjusted to a total volume of 900 µl by adding nuclease free water, and aliquots of 100 µl were pipetted into each fill port of a 384-well human LDA. Cards were centrifuged twice (12,000 rpm, 1 min, Multifuge3S-R, Heraeus, Germany), sealed, transferred into the 7900 RTQ-PCR instrument and a specific RTQ-PCR protocol was run over two hours using the 384-well LDA format.

The cycle threshold (CT) values (reflect the gene copy numbers) of the miRNAs of interest were normalized relative to the median miRNA expression value of all expressed miRNAs (Δ-CT approach). This allowed for a comparison of samples originating from different individuals which were processed at different times. For further analysis we calculated the ratio/fold change (FC) relative to the SHAM exposed samples (reference at the same time point) employing the ΔΔ-CT approach. A FC of one corresponds to a miRNA expression similar to sham-irradiated samples. A FC higher or lower than one refers to a several-fold over- or under-expression of the miRNA of interest after exposure relative to the reference. All technical procedures were performed in accordance to standard operating procedures (SOPs) implemented in the certification of the Bundeswehr Institute of Radiobiology according to DIN EN ISO 9001/2015. All materials used were ordered from Thermo Fischer/Applied Biosystems (Weiterstadt, Germany).

### Statistical analysis

Differential miRNA expression was analyzed as an outcome. In order to adjust for miRNA expression changes due to the exposure-time product and to identify temperature prone miRNAs we calculated the differential miRNA expression as followed.

- Differential miRNA expression of the control group was calculated with the ratio: normalized miRNA expression/mean normalized expression for each miRNA and donor. This calculation provided information about the inter-individual variance in miRNA expression of unexposed blood samples, enabling the identification of candidate miRNA with a lower inter-individual variance^13^.
- Differential miRNA expression of the EMF-exposure group was calculated relative to the SHAM exposure group at the corresponding exposure time with the ratio: normalized miRNA expression after EMF-exposure/normalized miRNA expression after SHAM exposure at the same time point for each transcript and donor. This calculation allowed correcting for miRNA expression changes occurring due to the blood storage in the EDTA tube over varying exposure time^13^.
- Differential miRNA expression of the RT + 2 °C-exposure group was calculated relative to the SHAM exposure group at the corresponding exposure time with the ratio: normalized miRNA expression after RT + 2 °C-exposure/normalized miRNA expression after SHAM exposure at the same time point for each transcript and donor. This calculation allowed to identify temperature prone miRNAs and to exclude these genes from the analysis^13^.

Descriptive statistics of continuous (n, mean, standard deviation, min, max) and categorical variables (frequency distributions) were performed and corresponding *p*-values (t-test as well as Chi-square/Fisher’s exact test, where applicable) were calculated for each of the miRNAs and per time point. We assessed the assumptions of normality (Kolmogorov–Smirnov) and equal variance and if required utilized either the pooled (equal variance) or the Satterthwaite variant of the t-test (unequal variance). Normality of miRNA expression values was fulfilled after log or boxcox transformation where necessary. Only those miRNAs that had a call “present” in at least 60% of RNA specimens were included in the analysis and only miRNAs with a *p*-value ≤ 0.05 among compared groups were selected as candidate miRNAs. For these miRNAs we finally examined whether their *p*-values will survive a Bonferroni correction for multiple comparison. Simple linear regression analyses were made for each donor and transcript to model the relationship between the differential miRNA expression relative to SHAM (dependent variable) and deposited electromagnetic energies (explanatory variable comprising the exposure-time product) in order to determine for dose–response relationships. All calculations were performed using SAS (release 9.2, Cary NC, USA)^15^.

### Ethical approval

The use of human peripheral blood cells (ex-vivo in vitro model) and the gene expression analysis of the screening study were approved by the ethics committee of the University of Ulm (Approval No. 371/14). Permission to conduct the validation study was given by the ethics committee of the Ludwig Maximilian University of Munich (Approval No. 18–588). We totally confirm that all research was performed in accordance with relevant guidelines and regulations. We also confirm that the informed consent was obtained from all participants.

## Results

### Peripheral blood cell counts and determination of the specific absorption rate (SAR)

In a previous publication on changes at the transcriptional level searching for protein coding messenger ribonucleic acids (mRNAs) and using the same biosamples we already reported about the normal values of differential blood cell counts observed in all donors^13^.

### Screening study from 2016 (n = 5)

#### Differential miRNA expression after exposure with EMF and hyperthermia

**Figure 2:**
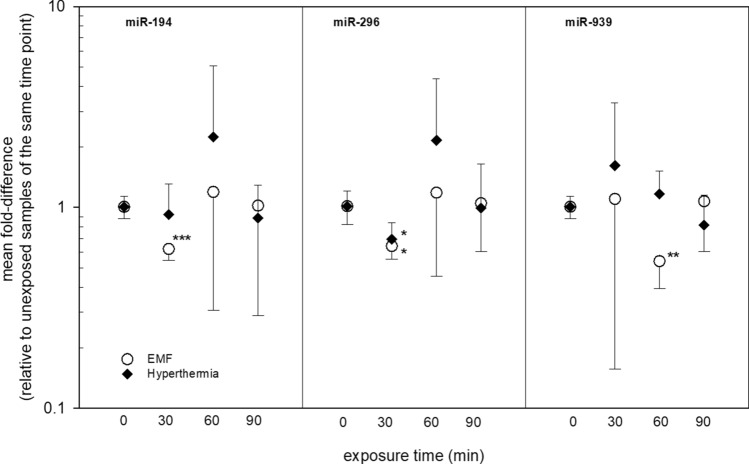
Provides mean fold-differences in miRNA gene expression of three miRNAs after EMF or hyperthermia exposure over the exposure time. Symbols represent mean fold-differences in gene expression and the error bars represent the SEM, n = 4–5. Asterisk reflects significance levels as follows: *p-value ≤ 0.05, **p-value ≤ 0.01, ***p-value ≤ 0.001.

**Figure 3:**
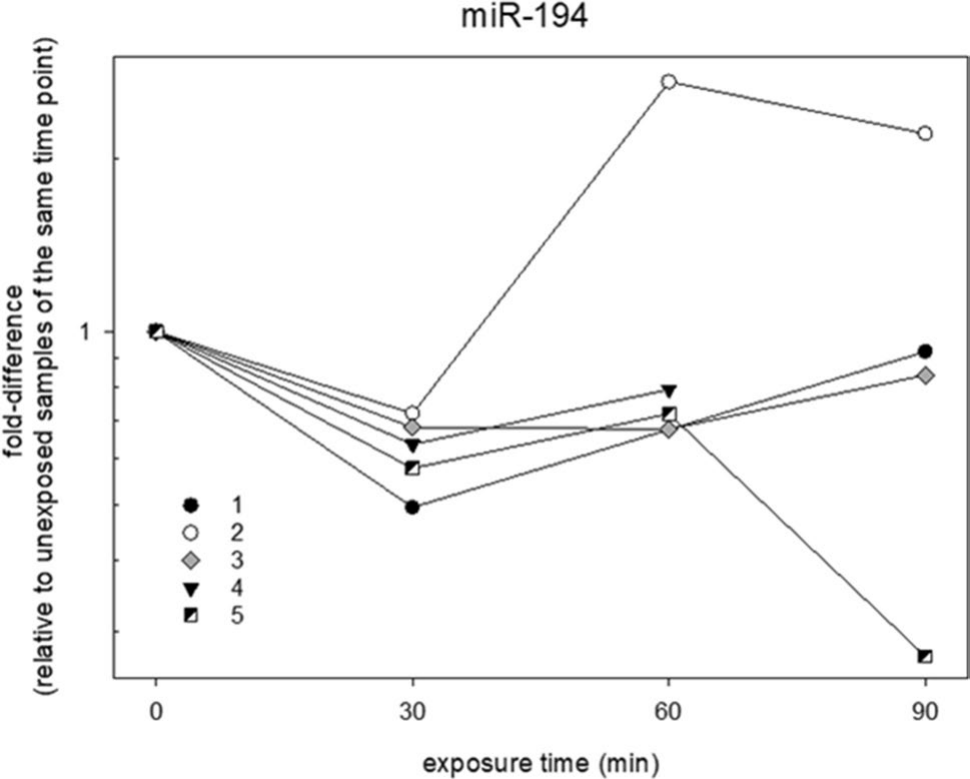
Reflects the individual fold-differences in miR-194 gene expression after EMF exposure of each donor over the exposure time which was only detected during the screening study in 2016.

Altogether 109 miRNAs from 667 miRNAs were eligible for analysis and nine of them (*miR-194, miR-296, miR-744, miR-324-3p, miR-532-3p, miR92a, mmu-miR-491, miR-664 and miR-939*) did show significant miRNA expression changes after EMF exposure. In particular *miR-194* at 30 min after EMF exposure appeared promising (Fig. 2). It was almost two-fold downregulated (FC = 0.62) and all five values showed a complete and significant (*p* = 0.0007) separation from the unexposed reference group (n = 4). Also, the p-value even survived the Bonferroni correction for multiple comparison (*p* < 0.0056, [*p* = 0.05/9]). The miR-939 showed similar features: a complete and significant (*p* = 0.004) separation of exposed from the unexposed reference group was observed and *miR-939* was almost two-fold downregulated (FC = 0.54) at 60 min after EMF exposure (Fig. 2). These significant miRNA changes after EMF exposure were not found after hyperthermia exposure. However, *miR-296* appeared significantly downregulated in the same way after 30 min EMF and hyperthermia exposure (Fig. 2). A significantly (*p* = 0.002) uneven frequency distribution of altered miRNAs over time after EMF exposure was found with seven out of nine miRNA significantly altered at 30 min, two at 60 min and none at 90 min after exposure (data not shown). Corresponding with that a significantly increased variance of all nine miRNAs over the exposure time was observed. By exemplifying the unequal variance, *miR-194* showed high congruence of miRNA expression for all donors only at 30 min, whereas different expressions occurred at all other points in time (Fig. 3).

**Figure 4:**
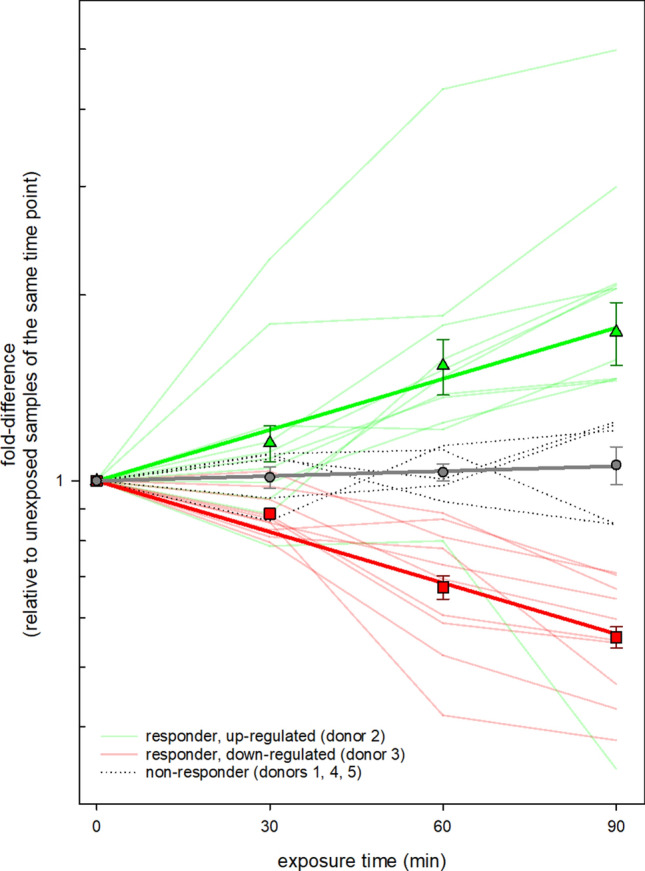
Displays the individual linear regression analysis of all donors in order to find miRNAs that were sufficient to demonstrate dose–response relationships. During the screening study 2016, donor 2 and donors 3 showed promising miRNA sets which correlated with exposure time (EMF energy deposition).

The observed unusual pattern of differentially expressed miRNAs over time as well as the inter-individual responses to EMF exposure-time products induced an alternative analysis approach. Here, we examined for miRNAs revealing an expected significant association of differential miRNA expression with the EMF exposure-time product, but in separate analyses for each donor. Altogether 11 up-regulated miRNAs (*miR-186, miR-222, miR-345, miR-486, miR-500, miR-20a, miR-20b, miR-339-5p, miR-425-5p, mmu-miR-93 and mmu-miR-374-5p*) and 10 down-regulated miRNAs (*miR-629, miR-660, miR-720, miR-106a, miR-151-5P, miR-18a-1, miR-30b, miR-324-3p, miR-886-5 and, mmu-miR-140*) appeared significantly associated with the EMF exposure-time product in donors 2 and 3, respectively (Fig. 4). Between 1 to 3 significantly deregulated miRNAs appeared associated with the EMF exposure-time products for the remaining donors 1, 4 and 5. The frequency distribution among the five donors was highly significant (*p* < 0.01), suggesting donors 2 and 3 to be in particular EMF-responsive.

### Validation study from 2018 (n = 5)

#### Comparing expressed miRNAs in 2016 with 2018

Altogether 667 miRNAs could be analyzed utilizing the LDA technology. In 2016 a number of 558 from 667 miRNAs appeared not expressed in the peripheral blood, leaving 109 miRNA species eligible for analysis. In 2018 almost the same miRNA species appeared expressed/not expressed and the overlapping number of similarly expressed/not expressed genes in both years was 97.8% (Table 2).

**Table 2:** Reflects the fourfold characterization of mean differential expressed and non-expressed miRNAs compared to SHAM from all donors in 2016 and 2018.

Reliability: 97,8%	2016		
	Expressed	Not-expressed	Sum
**2018**			
Expressed	95	1	96
Not-expressed	14	557	571
Sum	109	558	**667**

The analysis revealed a high degree of consistency concerning the expression behavior up to 97.8% by comparing both years. The total number (667) of all miRNAs examined in 2016 and 2018 is shown in bold.

#### Differential miRNA expression after exposure with EMF and hyperthermia

The mean statistic of all 5 donors showed now a missing deregulation of miR-194 after 30 min and miR-939 after 60 min.

**Figure 5:**
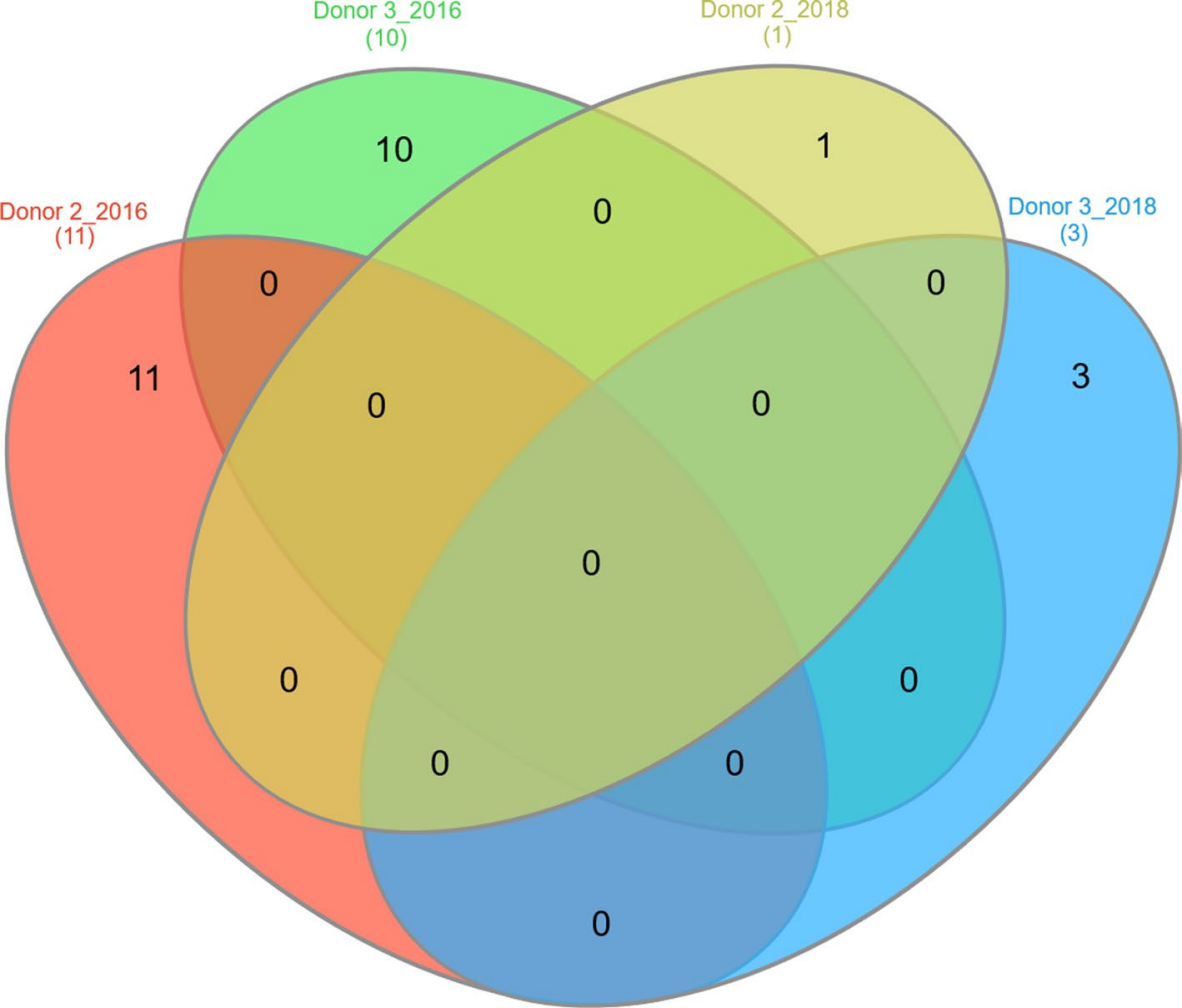
Shows the dose-dependent miRNA sets of donor 2 and donor 3 which have been identified to be potential EMF-responders during the screening study in 2016 compared with the number of dose-dependent miRNAs from the validation of the same donors in 2018. The venn diagram revealed the missing overlap of miRNA sets in both measurements.

When experimental processes were repeated with the same donors two years later, the number of significantly deregulated miRNA species decreased from 11 and 10 in 2016 to 1 (*miR-1233*) and 3 *(miR-144 and miR-99a*, *miR-331*) in 2018 for donors 2 and 3, respectively. None of the miRNA species showed an overlap in miRNA expression between 2016 and 2018 (Fig. 5). In contrast to 2016, there was no significant difference in the frequency distribution of deregulated miRNAs observed among the donors in 2018 (Table 2).

## Discussion

Based on our current assessment this is the first study that examines the athermal influence of 900 MHz EMF on miRNAs expressions in human peripheral blood cells. The coding sequences for miRNAs show a widespread distribution throughout the whole genome^16^ and act in its active state as fundamental biochemical control elements at the post-transcriptional level. Mice model knockout studies have shown that the inhibition of biogenesis from primary miRNA to the biological effective mature miRNA often straightly directs to embryogenic lethality^17,18^. Due to its crucial role in maintaining tissue homeostasis^19^, it has been speculated that athermal EMF might affect miRNA regulations.

We performed a 2-stage study design commencing with a screening study in order to formulate hypotheses in 2016 which were verified by a validation study using samples from the same 5 donors, two years later.

The screening study in 2016 revealed 2 promising candidates (miR-194 after 30 min and miR-939 after 60 min) that showed significant separation from control even after Bonferroni correction and were almost twofold down-regulated (Fig. 2). Nevertheless, a dose–response relationship that is considered to be a key criterion of causality as originally described by Bradford and Hill^20^, was absent through the mean statistical analysis of all donors for each of the tested 667 miRNAs. It was conspicuous that the miRNA expression variance of the mentioned candidates was predominantly high. An individual review of the miRNA expression values from miR-194 motivated the idea that an inter-individual miscellaneous response should also be taken into consideration (Fig. 3).

The distinctive characteristic or distinguishable degree of biological response in different individuals following low dose ionizing radiation was already postulated in previous studies^21^. It has been suggested that genetic or epigenetic predisposition and previous expositions are causally attributed to initiate inter-individual variance of the biological response after low dose irradiation.

Thus, linear regression analyses of each individual helped to identify possible relationships between the electromagnetic energy deposition (EMF exposure-time product) and miRNA expression. During the screening study 2016, we found the expected significant association of the EMF exposure-time product with correspondingly up- or downregulated miRNA species for 11 miRNAs in donor 2 and 10 miRNAs in donor 3 (Fig. 4). Donors 1, 4 and 5 showed only a small number of specific dose–response patterns. The significant differences in the observed frequency of differentially expressed miRNAs among donors stimulated to postulate donors 2 and 3 being more EMF-response, while donors 1, 4 and 5 were not.

The donor-dependent response pointed the way to repeat the experiments with the same donors after a temporal latency (here in about 2 years). During the validation study, the average analysis of all 5 donors was unable to confirm the downregulation of miR-194 after 30 min and miR-939 after 60 min as shown in the screening study.

Our study is therefore consistent with a whole series of earlier scientific investigations, which were initially able to show evidence of significant RNA expressions in radiofrequency EMF-exposed groups, but lacked to confirm these gene product deregulations in the subsequent validation study^22–25^. In contrast to the previous studies mentioned, the present work examined miRNA expressions instead of protein-coding mRNAs after radiofrequency EMF exposure. In general, published data on EMF exposure and the influence on miRNAs are very rare. To the best of our knowledge, there are only published studies that consider miRNAs and 50 Hz exposure from the energy supply. So Hualiang Li et al., described various miRNAs as biomarkers which could be isolated in exosomes from the serum of exposed mice in 2018. The authors concluded that different miRNAs are expressed depending on the magnetic flux density of the low frequency magnetic fields^26^. However, low frequency EMF shows a completely different interaction with biological systems and is ineligible to compare with a radiofrequency EMF exposure as applied to the peripheral blood cells in our current study.

In addition to the lack of evidence of the mean increased deregulation regarding miR-194 and miR-939, the candidate miRNAs being suspected of complying with a dose-expression relationship (donor 2 and donor 3), were also missing in the validation study (Fig. 4). However, methodological flaws appear unlikely, due to the comparison of all expressed and unexpressed miRNA-sequences from 2016 and 2018 which indicated a high degree of consistency up to 97.8% (Table 2).

These data suggest that the initially detected candidates belong to a random pattern due to the stochastic nature of gene regulation which is known to introduce some random noise into gene expression signatures^27^. It can be also argued that individual EMF-responsiveness does not persist over a period of 2 years and results could have been more reproducible when repeating the experiments within days or weeks. A suitable study design would be able to identify particular events in the life of the donors that could be related to an increased EMF-responsiveness. Also, it is unclear whether the time window chosen for examinations after exposure are sufficient for the induction of a biological response. For instance, several studies using ionizing radiation indicate an early transcriptional response to be detected earliest after 2–4 h^28,29^.

Irrespective of that, our results clearly underline the necessity to always perform a validation of miRNA expression readings.

In conclusion, the current study is the first examination with respects to the athermal impact of Radiofrequency EMF to the miRNA related posttranscriptional control in human peripheral blood cells. Our data suggest that 900 MHz EMF does not interfere with miRNA- regulation in human peripheral blood cells during EMF exposure up to 90 min. However, further studies observing alternative and commonly used frequencies with longer exposure times are needed to definitively state the independence of miRNA expression to EMF.

## Supplementary Information

Supplementary Information.

Supplementary Tables.
