# Supplementary material for: Analyzing the impact of 900 MHz EMF short-term exposure to the expression of 667 miRNAs in human peripheral blood cells
Source: Sci Rep. 2021 Feb 24;11:4444. doi: 10.1038/s41598-021-82278-1 (PMC7904780; doi:10.1038/s41598-021-82278-1)
Supplement: Supplementary file 2 — Supplementary Tables. [file 41598_2021_82278_MOESM2_ESM.pdf]

Analyzing the impact of 900 MHz EMF short-term exposure to the expression of 667 miRNAs in human peripheral blood cells

Andreas Lamkowski, Matthias Kreitlow, Jörg Radunz, Martin Willenbockel, Marcus Stiemer, Lars Ole Fichte, Carl Friedrich Rädel, Matthäus Majewski, Patrick Ostheim, Matthias Port and Michael Abend

Supplemental table description

Data regarding RNA-isolation, raw and normalized Ct-values as well as fold-change calculations are provided for replicate measurements on five healthy donors performed in 2016 and 2018 in separate folder of one excel data file. Code descriptions are implemented where used for the first time. Instead of showing raw-Ct-values we provided the normalized Ct-values and the housekeeping genes (median Ct-values – 15 to avoid negative Ct-values). Median values differ between LDA (low density arrays, numbered from 1 to 50) and are presented for LDA type A and B, separately.
